# Fusion of Multimodal Spatio-Temporal Features and 3D Deformable Convolution Based on Sign Language Recognition in Sensor Networks

**DOI:** 10.3390/s25144378

**Published:** 2025-07-13

**Authors:** Qian Zhou, Hui Li, Weizhi Meng, Hua Dai, Tianyu Zhou, Guineng Zheng

**Affiliations:** 1 School of Computer Science, Nanjing University of Posts and Telecommunications, 9 Wenyuan Road, Nanjing 210023, China; zhouqian@njupt.edu.cn (Q.Z.); 1222045824@njupt.edu.cn (H.L.); daihua@njupt.edu.cn (H.D.); 2 School of Ccomputing and Communications, Lancaster University, Lancaster LA1 4YR, UK; 3 School of Modern Posts, Nanjing University of Posts and Telecommunications, Nanjing 210003, China; 1224097337@njupt.edu.cn; 4 School of Computing, University of Utah, 50 S. Central Campus Dr. Rm. 3190, Salt Lake City, UT 84112-9205, USA; guineng@cs.utah.edu

**Keywords:** sign language recognition, multimodal fusion, spatio-temporal graph convolutional network, ResNet2+1D

## Abstract

Sign language is a complex and dynamic visual language that requires the coordinated movement of various body parts, such as the hands, arms, and limbs—making it an ideal application domain for sensor networks to capture and interpret human gestures accurately. To address the intricate task of precise and expedient SLR from raw videos, this study introduces a novel deep learning approach by devising a multimodal framework for SLR. Specifically, feature extraction models are built based on two modalities: skeleton and RGB images. In this paper, we firstly propose a Multi-Stream Spatio-Temporal Graph Convolutional Network (MSGCN) that relies on three modules: a decoupling graph convolutional network, a self-emphasizing temporal convolutional network, and a spatio-temporal joint attention module. These modules are combined to capture the spatio-temporal information in multi-stream skeleton features. Secondly, we propose a 3D ResNet model based on deformable convolution (D-ResNet) to model complex spatial and temporal sequences in the original raw images. Finally, a gating mechanism-based Multi-Stream Fusion Module (MFM) is employed to merge the results of the two modalities. Extensive experiments are conducted on the public datasets AUTSL and WLASL, achieving competitive results compared to state-of-the-art systems.

## 1. Introduction

With the rapid advancement of sensor technology, wearable sign-language capture devices based on inertial measurement units (IMUs) and bending sensors can collect dynamic motion data from the hands, limbs, and facial expressions in real time [1]. These rich motion signals, once processed through machine-learning models, enable high-precision sign-language recognition and translation. Such sensor-driven sign language recognition systems have already found applications in smart home control [2], in remote medical communication, and as teaching aids for sign language learners, bridging the communication gap between people with hearing impairments and the hearing community. Sign language is a complex and dynamic visual language that requires the coordinated movement of various body parts, such as the hands, arms, and limbs—making it an ideal application domain for sensor networks to capture and interpret human gestures accurately. There are two primary challenges for sign language recognition (SLR) [3]. Firstly, SL involves the cooperative representation of multiple body parts, resulting in high spatial complexity. Secondly, as a dynamic language, SL requires consideration of the temporal interplay between sequential actions.

SLR can be categorized into sensor-based and vision-based methods according to data acquisition approaches. Early studies mainly used wearable devices such as data gloves [4], which offered high accuracy but were inconvenient to wear, while vision-based methods have recently gained more attention due to their non-contact and low-cost advantages. Nevertheless, vision-based SLR is highly sensitive to environmental factors such as lighting, occlusion, and background complexity. In contrast, sensor-based approaches can provide structured, noise-robust motion signals but often lack the rich visual information critical for differentiating similar gestures. To overcome these limitations, the integration of multimodal sensor systems—combining both physical sensors (e.g., IMUs, data gloves) and virtual sensors (e.g., computer vision-based pose estimation)—has emerged as a promising direction for creating robust and adaptive SLR systems. In this context, visual skeleton estimation algorithms effectively serve as non-contact soft sensing modalities, augmenting RGB video data by capturing structured spatio-temporal representations of gestures. This sensor fusion paradigm aligns closely with the objectives of intelligent sensing research, where diverse sensing modalities are combined to enhance perception accuracy and resilience.

Early vision-based SLR approaches primarily relied on image processing techniques and various methods for extracting visual features. As machine learning techniques have boomed, they are increasingly employed to model the SLR problem. Initially, visual features of sign language were extracted using methods such as Scale-Invariant Feature Transformation (SIFT) [5] or Histogram of Oriented Gradients (HOG) [6], which were then employed to train models such as Support Vector Machines (SVMs) or Random Forests [7]. Moreover, Hidden Markov Models (HMMs) and their extensions have demonstrated efficacy in modeling temporal data, becoming staples in SLR applications. For instance, adaptive HMMs [8] have been tailored to construct individual learning models for each sign language word, enhancing SLR accuracy.

With the advent of deep learning, fields such as computer vision and pattern recognition have witnessed substantial advancements, thereby propelling SLR research forward. Techniques like the Long Short-Term Memory Network (LSTM) [9], renowned for its robust capability in sequence data modeling, and the 3D convolutional neural network (3D CNN) [10], effective in target detection, have been progressively integrated into SLR studies. Further innovations include the adaptation of the 3D Convolutional Residual Network (3D ResNet) [11] to extract spatio-temporal features relevant to SLR and the incorporation of attention mechanisms within 3D CNN frameworks to isolate and emphasize critical local features [12]. Although some studies have utilized contact-based sensors such as IMUs and data gloves to capture sign language data for deep learning models, these methods face limitations in terms of user comfort and practical deployment. Most current methods are still based on RGB videos for model construction, which are easily affected by lighting, viewing angles, and complex backgrounds, often resulting in decreased recognition accuracy. In recent years, the use of human joint estimation based on vision has been widely explored. Joint positions can effectively compensate for the limitations of RGB data, and Spatio-Temporal Graph Convolutional Networks (ST-GCNs) have been employed to extract spatial and temporal features from the generated skeleton sequences efficiently.

In this paper, we introduce a multimodal learning approach for SLR leveraging both skeleton and raw RGB data. Our model comprises three main components: (1) A novel Multi-Stream Spatio-Temporal Graph Convolutional Network (MSGCN), which adopts a multi-stream structure to enhance feature extraction. An MSGCN unit integrates an decoupling graph convolutional network (decoupling GCN) for spatial data handling. A self-emphasized temporal convolutional network (SETCN) is used to model temporal data processing, and a novel spatio-temporal joint attention module (SJAM) is proposed to highlight important spatio-temporal features in SLR. (2) A 3D ResNet network based on deformable convolutions, combining a ResNet2+1D network and a 3D deformable convolution (D-ResNet), is proposed to adaptively capture complex spatial and temporal features in raw RGB videos. (3) An end-to-end sensor fusion framework that synergistically integrates skeleton and RGB data to improve recognition robustness and accuracy. By framing our approach as an intelligent visual sensing system that unifies raw video and virtual skeleton representations, we aim to address the challenges of dynamic gesture recognition in uncontrolled environments. Extensive experiments on the AUTSL and WLASL datasets show that our method achieves results comparable to current state-of-the-art techniques. The rest of the paper is organized as follows. Section 2 provides a brief overview of related research. Section 3 describes the details of the proposed bimodal sign language recognition-based framework. Section 4 presents the experimental setup and experimental results of the model on the AUTSL dataset and WLASL dataset. Section 5 gives conclusions and discussion.

## 2. Related Work

### 2.1. Raw Image-Based SLR

Sign language recognition (SLR) based on raw image inputs typically utilizes RGB video frames to extract visual appearance and motion features. These visual cues are processed using 2D/3D convolutional neural networks (CNNs), often complemented by recurrent neural networks (RNNs) or transformers to capture temporal dependencies. The raw video data is captured through conventional cameras or RGB sensors, and models learn to extract spatial features (e.g., hand shapes, body posture) and temporal dynamics (e.g., motion trajectories) directly from image sequences. While these methods benefit from rich visual information, they are often sensitive to lighting, background clutter, and occlusions.

Traditional SLR includes SVMs on SIFT features [13], temporal modeling based on HMM [14], and routine classification-based trajectory modeling [15]. Recent advancements in deep learning have transformed SLR using CNN and RNN [16,17,18], where CNNs and RNNs excel at extracting spatial and temporal features, respectively. Innovations include Liu et al.’s end-to-end LSTM model [19], Masood et al.’s use of CNNs and RNNs [20], and Basnin et al.’s superior CNN-LSTM integration [21]. Furthermore, 3D CNNs, which manage both spatial and temporal features, have been enhanced by Huang et al. [12] with attention mechanisms and combined by Bendarkar et al. [22] with bi-directional LSTMs. Yang et al. [23] merged 2D with 3D CNNs in their Sf-net model, and Hu et al. [24] incorporated local hand information into a deep residual network (ResNet). Despite these advances, the dependency on unstable RGB images compromises model accuracy and robustness, prompting this paper to explore alternative modalities to improve SLR models.

### 2.2. Skeleton-Based SLR

Skeleton-based SLR methods rely on pose estimation algorithms (e.g., OpenPose, HRNet, MediaPipe) to extract joint keypoints of the human body from RGB or depth videos. These joints represent the signer’s posture over time and can be used to compute motion trajectories, body structure, and gesture patterns in a compact and invariant form. Such data is especially suitable for spatio-temporal modeling using graph convolutional networks (GCNs), due to its structured nature.

Recent studies have leveraged skeleton data for SLR. Bohacek et al. [25] detected the 2D sign joint coordinates using skeletons, applied data augmentation, and used a temporal model for SLR. Liu et al. [26] developed a multi-channel model based on skeleton joints, and Coster et al. [27] enhanced skeleton joint data with Gaussian noise before initiating SLR. Graph convolutional networks (GCNs) have proven effective in sketching skeleton maps. Tunga et al. [28] used a GCN to process skeleton maps for spatial feature extraction, while Parelli et al. [29] combined a GCN with BiLSTM to model SLR from various skeleton features. Meng et al. [30] proposed a multi-scale GCN approach to extract spatio-temporal features on multiple scales. Naz et al. [31] utilized MediaPipe for joint extraction, merging spatial and temporal convolutions in GCN. Guo et al. [32] proposed a Hand Shift Decoupling GCN module to explore the correlation between remote joints for more accurate SLR. Miah et al. [33] developed a multi-stream GCN module to process the four types of skeleton features, enhancing SLR through feature splicing. Despite their effectiveness, challenges remain in capturing remote spatial correlations and complex temporal patterns in SLR.

### 2.3. Multimodality-Based SLR

Multimodal SLR methods aim to fuse complementary information from multiple data sources—such as RGB, depth, skeleton, and optical flow—extracted via diverse sensors like depth cameras (e.g., Kinect), RGB sensors, and wearable devices. This fusion strategy helps compensate for the weaknesses of individual modalities and improves robustness under various environmental conditions.

For SLR, multi-feature integration involves both single-modality and multimodality approaches. Multimodal fusion, increasingly popular in action recognition, combines various data types like RGB images, depth images, optical flow, and skeletons to improve model accuracy. For example, Gao et al. [34] used a dual-stream CNN to process RGB and depth images, merging them for enhanced recognition. Xiao et al.’s [35] CRB-Net and Rastgoo et al.’s [36] approach similarly integrate multiple data types for SLR, using architectures like BiLSTM and LSTM for processing. Ref. [37] introduced a multi-stream CNN integrating depth information, RGB color, and image features to improve the model. In addition, some strategies focus on extracting multiple features from a single modality. Almeida et al. [38] extracted multiple visual features from depth images. Wang [11] and Gokcce et al. [39] utilized RGB images for similar purposes, employing models like 3D ResNet and fusion networks to maximize the data’s utility. Jiang et al. [40] extracted six features from RGB and RGB-D images, including optical flow and Horizon Height Angle, to enhance feature extraction. Hu et al. [41] proposed a self-supervised pre-training framework, SignBERT+, which incorporates model-aware hand priors and multi-level masked modeling to enhance sign language understanding, achieving state-of-the-art performance in both recognition and translation tasks. Zhang et al. [42] extracted spatio-temporal features and complementary information from different modalities by constructing a multi-level and multi-view spatio-temporal detector and achieved efficient multimodal feature fusion and redundancy suppression through an attention mechanism combined with Canberra distance. In addition, Xu et al. [43] proposed a multimodal recognition framework based on deformable convolutional networks that adaptively learn sampling offsets and feature amplitudes to better capture prominent spatio-temporal patterns in heterogeneous modalities.

## 3. Proposed Method

**Figure 1:**
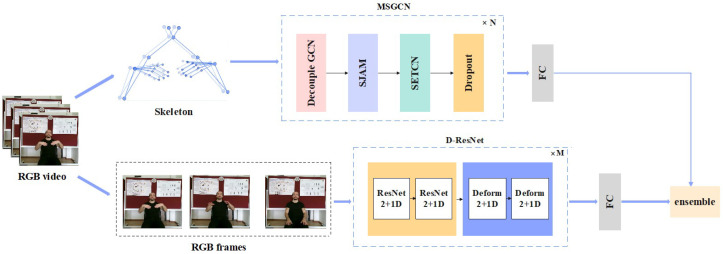
An illustration of the proposed dual-path sign language recognition framework: the MSGCN path extracts multi-stream skeleton features using spatio-temporal graph convolution to capture spatial and temporal dependencies in skeletal data, while the D-ResNet path employs deformable 3D convolutions to adaptively model complex spatial and temporal variations in raw RGB videos. The outputs from both paths are fused through a gate-based late fusion mechanism to produce the final prediction.

In this study, we introduce a dual-modality SLR method that integrates data from both skeleton and RGB image modalities. The overall architecture is shown in Figure 1. The first modality employs a Multi-Stream Spatio-Temporal Graph Convolutional Network (**MSGCN**) model to process both temporal and spatial dimensions of skeleton data. The second modality utilizes a deformable convolution-based 3D ResNet (**D-ResNet**) model to analyze spatial and temporal patterns from raw images. A late fusion layer combines the outputs of these two pathways to enhance the overall performance of the model.

### 3.1. Skeleton Modality

For our SLR system, we firstly use the HRNet whole-body pose estimation network [44] to extract keypoint data from RGB videos. HRNet can detect 133 keypoints across the entire body. However, not all of them relate to SLR. In this work, we select 27 nodes, which include 10 nodes for each hand and 7 nodes for the upper body. In addition, dynamics of hand gestures can also be used to capture the beneficial information. To exploit this, we adopt a multi-stream structure that enriches the input to our model, encompassing joint features, joint motion features, bone features, and bone motion features. We then employ an MSGCN model to process the skeleton data, utilizing a decoupling GCN, a self-emphasizing temporal convolutional network, and a spatio-temporal joint attention module specifically suited for SLR.

#### 3.1.1. Multi-Stream Skeleton Features

**Figure 2:**
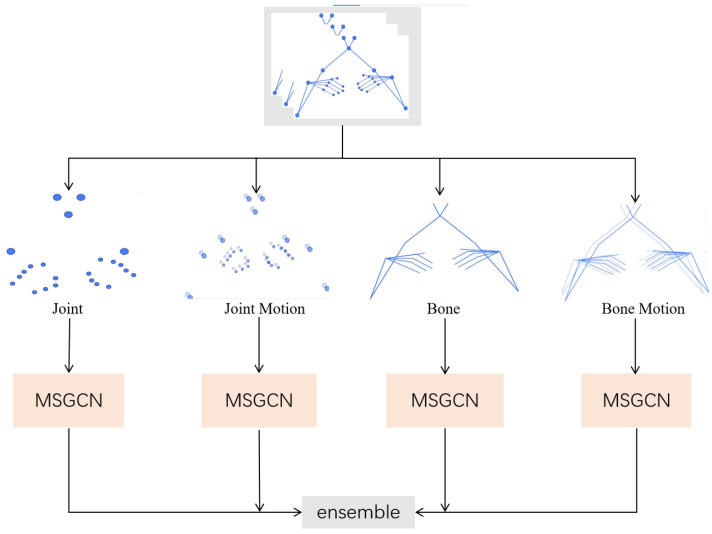
An illustration of the fusion of multi-stream skeleton features: the skeleton data of the four streams are fed into the MSGCN model separately for processing, and the resulting features are fused.

Traditional skeleton-based methods rely solely on basic joint coordinates, which often lack sufficient information about motion dynamics and the structural characteristics of sign language. Inspired by the findings of Shi et al. [45], we recognize that incorporating additional features and designing a multi-stream architecture can significantly enhance the model’s capacity to accurately recognize complex sign language gestures. To this end, we extract four skeleton features—joint features, joint motion features, bone features, and bone motion features—to build a multi-stream structure. Joint features are represented by the coordinates of nodes, denoted as $J_{i,t}=x_{i,t},y_{i,t},s_{i,t}$, where *x*, *y* are the horizontal and vertical coordinates and *s* is the confidence score. In HRNet, this confidence score is computed based on the heatmap response at each joint location, where each pixel in the heatmap represents the probability that a keypoint exists at that position. This enables the model to assign spatial certainty to detected joints. The joint features represent the positional information of the gesture in space. Joint motion features reflect the temporal change in node positions and are calculated as $JM_{i,t}=J_{i,t+1}-J_{i,t}$. Bone features are derived from the natural connections of the human body, represented by the vector between a source node and its adjacent target node and calculated as $B_{i,t}=J_{i,t}-J_{k,t}$, which encodes relative spatial relationships and possesses rotation invariance. Bone motion features reflect the temporal variation in bone vectors and are calculated as $BM_{i,t}=B_{i,t+1}-B_{i,t}$. As shown in Figure 2, by extracting these four types of features and structuring them into a multi-stream framework, our model can more comprehensively capture and interpret complex SLR movements, thereby enhancing recognition accuracy. 

#### 3.1.2. MSGCN

**Decoupling GCN.** The traditional spatial graph convolutional network (GCN) has certain limitations; specifically, all channels in the input feature *X* share the same adjacency matrix *A* and the same feature transformation matrix during spatial aggregation. This sharing mechanism makes it difficult for the model to capture correlations among different body parts. Following the insight of Chen et al. [46], we address this limitation by employing a decoupled GCN, which assigns independent adjacency matrices to different channels. This allows each channel to perform spatial aggregation independently, enabling more flexible modeling of structural relationships between body parts. However, this approach can lead to parameter redundancy and potential overfitting. To mitigate this, we introduce a graph descent module within the network to compress the parameter space effectively and enhance the model’s generalization ability. The computation process is formulated as shown in Equations (1) and (2).

(1) $$X_{\mathrm{out}}={\tilde{A}}_{1}X_{0:[\frac{C}{g}]}W∥{\tilde{A}}_{2}X_{\left[\frac{C}{g}\right]:\left[\frac{2C}{g}\right]}W∥...∥{\tilde{A}}_{g}X_{[\frac{C}{g}]:C}W\;$$

$$Drop=\frac{1-Prob_{keep}}{Drop_{size}}$$

where *X* denotes the input and *A* is the adjacency matrix. To ensure self-connections, the normalized adjacency matrix is computed as $\tilde{A}=A+I$, where *I* is the identity matrix. This allows each node to aggregate its own features along with its neighbors’. *g* is the number of adjacency matrices, and *X* is divided into *g* groups, each of which has a separate adjacency matrix and a feature transformation matrix aggregating the features of the nodes. ∥ is a join operation that combines the aggregated results of the *g* groups. Drop represents the dropout probability of regions in the graph descent module, which is calculated based on the keep probability $Prob_{keep}$ and the number of dropped nodes $Drop_{size}$.

**Self-emphasizing TCN.** Temporal information is also important for SLR, especially given the dynamic nature of sign language. Traditional methods often utilize a 1D temporal convolution network (TCN), which treats each frame equally without considering the varying significance of different frames in a sign language video. Recognizing that not all frames contribute equally to the interpretation of signs, with some frames being merely transitional and others being pivotal for understanding the message, our method introduces a self-timing emphasis module: a self-emphasized TCN (SETCN). It adaptively highlights key frames while diminishing the impact of less informative or transitional frames.

**Figure 3:**
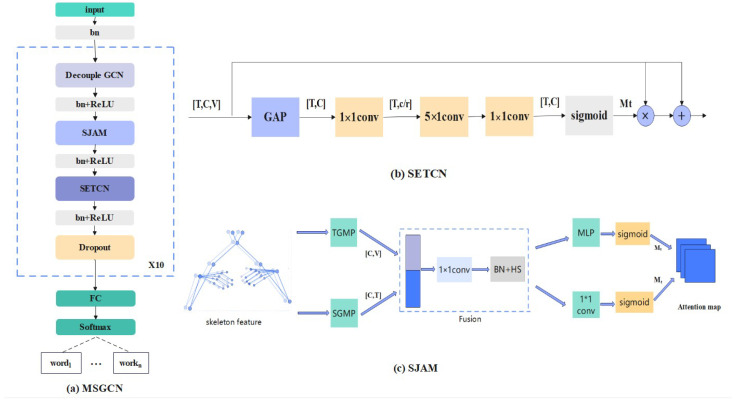
An illustration of the MSGCN architecture: (**a**) a schematic diagram of the MSGCN unit network details; (**b**) the self-emphasized temporal convolution module; (**c**) the spatio-temporal joint attention module.

The specific implementation of the SETCN module is shown in Figure 3b. It firstly uses global average pooling and 1×1 convolution to reduce the dimensions of feature and channel, obtaining the feature vector $X_{p}$. Then, a 5×1 convolution is used to capture the temporal information in the sequence. Afterwards, the processed temporal features are fed into the 1×1 convolution to recover the channel dimensions. Finally, a sigmoid function is applied to generate the attention graph $M_{t}$. It adaptively emphasizes key frames within the time series while suppressing useless frames and transitional frames. To preserve the integrity of the original feature information and mitigate issues related to gradient vanishing, residual concatenation is employed. SETCN is formalized in Equations (2)–(5).

(2) $$X_{p}=Conv_{k=1}\left(GAP\left(X_{\mathrm{in}}\right)\right)\;$$

(3) $$X_{t}=Conv_{k=1}\left(Conv_{k=5}\right)\;$$

(4) $$M_{t}=Sigmoid\left(X_{t}\right)\;$$

(5) $$X_{\mathrm{out}}=M_{t}⊙X_{\mathrm{in}}+X_{\mathrm{in}}\;$$

where $X_{\mathrm{in}}$ denotes the input to the SETCN module, GAP denotes global average pooling, *k* denotes the convolutional kernel size, $X_{p}$ denotes the processed intermediate feature vector, $M_{t}$ denotes the attention maps, ⊙ denotes the multiplication in the elemental direction, and $X_{\mathrm{out}}$ is the final output.

In the context of temporal modeling for SLR, some research methods have explored the use of multi-scale TCN modules. These modules are designed to capture timing information at various temporal resolutions by constructing TCN branches with differing scales. However, in our investigation, we find that these operations tend to complicate the model. This increased complexity does not necessarily compensate the prediction accuracy. Our proposed model not only simplifies the model structure but also targets the most relevant temporal features more directly and effectively.

**Spatio-temporal joint attention module.** An attention mechanism is introduced to enhance the extraction of spatio-temporal features by our model. Attention modules constructed in traditional models usually perform feature enhancement according to specific dimensions, such as executing the attention modules in spatial, temporal, and channel dimensions independently. However, according to the characteristics of sign language, spatial information and temporal information are related to each other. Based on this, we propose a spatio-temporal joint attention module SJAM for SLR based on the work of Song et al. [47]. We extract features based on the spatio-temporal information, enhance the spatio-temporal features separately, and finally fuse them to obtain the spatio-temporal joint attention map, which enhances the key spatio-temporal information of the skeleton and captures the dynamic spatio-temporal features of the skeleton effectively.

As shown in Figure 3c, firstly, two feature vectors are obtained by global average pooling in the temporal and spatial dimensions, they are spliced in the channel dimension for spatio-temporal feature fusion, and a 1×1 convolution is used to reduce the channel dimension. Next, two independent attention modules are constructed to enhance the temporal and spatial dimensions, respectively. Finally, a weighted fusion is performed to obtain a joint spatio-temporal attention map to realize the enhancement of key spatio-temporal features. The output of the SJAM is given by Equations (6)–(10).

(6) $$X^{\prime }=TGMP\left(X_{\mathrm{in}}\right)⊕SGMP\left(X_{\mathrm{in}}\right)\;$$

(7) $$X^{\prime \prime }=HS\left(Conv_{k=1}\left(X^{\prime }\right)\right)\;$$

(8) $$M_{t}=Sigmoid\left(Conv_{k=1}\left(X^{\prime \prime }\right)\right)\;$$

(9) $$M_{v}=Sigmoid\left(MLP\left(X^{\prime \prime }\right)\right)\;$$

(10) $$X_{\mathrm{out}}=\left(M_{t}⊗M_{v}\right)⊙X_{\mathrm{in}}\;$$

where $X_{\mathrm{in}}$ is the input to the SJAM module. TGMP and SGMP denote global max-pooling in the time dimension and global max-pooling in the spatial dimension for in-channel splicing operations, HS denotes the HardSwish activation function, $M_{t}$ and $M_{v}$ denote the attention maps in the temporal and spatial dimensions, respectively, ⊗ denotes the out-of-channel multiplication, and ⊙ denotes the elemental multiplication.

In summary, the basic unit of the MSGCN mainly consists of decoupling GCN, SJAM, and SETCN. A batch normalization layer and a ReLU activation function layer are added after each module. The use of residual connections between the modules can make the training more stable. The MSGCN module, which consists of 10 basic units, is adopted successively. Finally, a linear connection layer is applied to achieve the standard multi-class classification using the softmax activate function. The process is shown in Figure 3a.

### 3.2. Raw Image Modality

A raw RGB image itself contains a lot of spatio-temporal information. Traditionally, 3D CNNs have been employed to extract these features for SLR. But they are computationally intensive and prone to overfit. To solve this problem, we propose a deformable convolution-based 3D ResNet (**D-ResNet**), which uses a ResNet2+1D network [48] as the basic architecture of the model. Compared with 3D CNNs, ResNet2+1D has the advantage of its ability to decompose spatio-temporal features into 2D spatial features and 1D temporal features, and this decomposition is able to both effectively capture the video’s spatio-temporal information and at the same time make the model more lightweight and reduce the computational cost. In addition, deformable convolution [49] can dynamically adjust the shape and position of the convolution kernel, improving adaptability to variations in movement and background, thereby enhancing SLR accuracy. So, we combine 3D deformable convolution in the last two layers of the model to improve the accuracy of SLR recognition.

**Figure 4:**
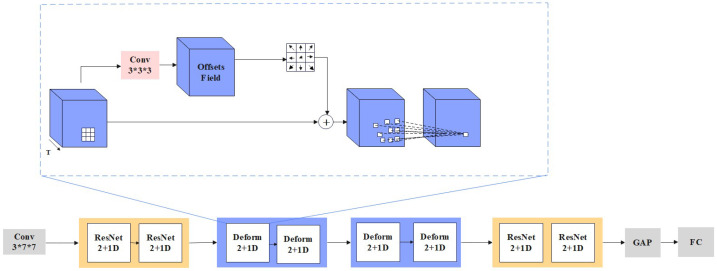
Illustration of proposed 3D deformable convolution for adaptive spatio-temporal learning. Notes: only deformable spatial convolution is illustrated here; deformable temporal convolution is similar.

**3D deformable convolution.** Three-dimensional deformable convolution can adaptively capture spatio-temporal features on raw RGB video. Its structure is shown in Figure 4, specifically, deformable 3D convolution achieves spatial deformation and temporal deformation by introducing additional parameterized offsets. The deformable 3D layer adopts an offset convolution layer to generate the offsets and then feeds the feature map X into the offset convolution layer to obtain the offset features. The learned offsets are used to guide the sampling network to generate the deformable sampling network, and finally the deformable sampling network outputs the extracted features. According to the characteristics of the ResNet2+1D network, the deformable 3D network is divided into two parts, firstly a deformable spatial convolution on 2D spatial features and secondly a deformable temporal convolution on 1D time series. Its computational process is shown in Equations (11) and (12).

(11) $$Y=DeConv(X,W_{H\times W},f_{H\times W}\left(X\right))\;$$

(12) $$Z=DeConv(Y,W_{T},f_{T}\left(Y\right))\;$$

where *X* denotes the input, while *Y* is the intermediate feature vector produced after processing through the network. Parameters $W_{H\times W}$ and $W_{T}$ correspond to the weights of the deformable spatial convolution and the deformable temporal convolution, respectively. These deformable convolutions allow the model to adapt more effectively to variations in both spatial and temporal dimensions by modifying the convolution kernels based on the computed offsets $f_{H\times W}$ and $f_{T}$, which represent the spatial and temporal offsets, respectively.

In addition, due to the large number of parameters in the model, the Swish activation function is employed instead of the traditional ReLU. The Swish function is known for promoting smoother gradient flow during backpropagation, which can enhance the convergence speed and improve the generalization capabilities of the model.

### 3.3. Multimodal Fusion

In the final stage of our model, we employ a Multi-Stream Fusion Module (MFM) that uses a gating mechanism to combine the outputs from different streams into one prediction. Each individual stream processes the input through their own network. Their outputs are denoted as $q_{i}$, where *i* denotes the specific stream fused into the final output.

The fusion process includes the following stages: **Channel compression:** the channel dimensions of each stream $q_{i}$ are compressed to reduce the dimensions and complexity of the output, making it more suitable for the subsequent gating operation. **Gating:** The compressed results are then fed into a gating unit, which computes a gating value $g_{i}$ for each stream. Gating values serve to determine the relative importance of each stream’s output in the final prediction. **Weighted summation:** Each stream’s output is then multiplied by its corresponding gating value $g_{i}$. It produces a set of weighted results, one for each stream. Those weighted results are summed together to form the final prediction output. This process is shown in Equations (13) and (14):

(13) $$g_{i}=\sigma \left(W_{g}.q_{i}+b_{g}\right)\;$$

(14) $$y=\sum_{i=1}^{n}\left(g_{i}.q_{i}\right)\;$$

where $g_{i}$ denotes the weight of the ith stream, σ is the Sigmoid function, $W_{g}$ and $b_{g}$ are learnable parameters, and *y* denotes the final result. In such a way, the MFM can dynamically adjust the weights according to the importance of different streams, thus improving the overall model performance.

## 4. Experiments and Results Analysis

In this section, we conduct extensive experiments on two public datasets, AUTSL and WLASL, and compare them with state-of-the-art research methods. In this section, we present the datasets, implementation details, comparative experimental results, and ablation experimental results in turn.

### 4.1. Evaluation Datasets

The Ankara University Turkish Sign Language (AUTSL) dataset [50] is a Turkish Sign Language dataset that contains multimodal data including RGB videos, skeletal data, and depth data. It comprises 226 commonly used Turkish sign language words, totaling 36,302 samples. In this study, we utilize only the RGB video modality. The dataset was recorded by 43 participants across 20 different natural environments. Each video has a resolution of 512 × 512 pixels and is recorded at 30 frames per second. All experiments follow the official training, validation, and test splits provided with the dataset.

The Word-level American Sign Language (WLASL) dataset [51] is the largest video dataset currently available for word-level American SLR and is intended to facilitate research in word-level SLR. The dataset contains 2000 common words in American Sign Language with a wide range of word coverage and a total of 21,083 RGB videos recorded in different contexts by 119 recorders of different statures. The official dataset is further classified into WLASL-100, WLASL-300, WLASL-1000, and WLASL-2000, and currently the accuracy of WLASL-2000 is low, and it is a challenging dataset; in this paper we use WLASL-2000 for our experiments.

In addition to the above datasets, we also explored other word-level sign language datasets, as shown in Table 1. Previous studies have shown that the LSA64 dataset has achieved 100% correctness and the CSL-500 dataset has 99% accuracy. Since the BSL-1K dataset is not publicly available, the AUTSL and WLASL datasets are chosen for experiments in this paper.

### 4.2. Implementation Details

This subsection describes the experimental setup in detail. In the skeleton-based model implementation, the skeleton data is first extracted from the original RGB video using the HRNet whole-body pose estimation network provided by MMPose; a total of 27 keypoints of the left and right hand and the upper limbs are used to construct the spatio-temporal skeleton map; and random sampling, rotation, and scaling are performed to perform data enhancement. During the training process, the batch size is set to 64; the Adam optimizer is used; the initial learning rate is set to 0.1 and weight decay to 0.0001 for a total of 180 iterations; and the optimization phase is set to 0.01 for the initial learning rate, 0.0001 for the weight decay, and 50 iterations. In addition, since the number of samples corresponding to each label in the WLASL-2000 dataset is too small to be adequately trained, the model parameters obtained from training on the AUTSL dataset are used as the pre-trained model for training on the WLASL dataset, with a total of 100 rounds of iterations. Top-1 and Top-5 accuracy were used as evaluation metrics on the dataset.

In the model implementation based on RGB images, the video samples are randomly sampled by openCV to extract 32 frames of RGB image sequences, then the pictures are scaled to 256 × 256, and the symbolic image sequences are augmented with data such as random horizontal inversion and mirroring. The batch size of the model training process is set to 8. Using the Adam optimizer, the initial learning rate is set to 0.001, the weight decay is 0.0001 for 100 iterations, and the optimization phase is set to 0.0001 for the initial learning rate, 0.0001 for the weight decay, and 100 iterations. In addition, due to the large number of model parameters, we first use the CSL-500 dataset for pre-training.

Finally, the results of the two models were fused together at a later stage. All experiments were conducted using the PyTorch 1.11 deep learning framework. The experimental environment was built on a high-performance computing server running the Ubuntu 20.04 LTS operating system. The server is equipped with an Intel Core i7-12700KF processor featuring eight physical cores and sixteen threads, 64 GB of DDR4 memory, and two Nvidia RTX 4090 GPUs with 24 GB of VRAM each. GPU acceleration is supported via CUDA 11.3, enabling efficient training of deep learning models. The programming environment is based on Python 3.8, managed through Anaconda 23.7.4, which ensures stability and comprehensive support for the required scientific computing libraries.

### 4.3. Performance Comparison

To assess the performance of our multimodal SLR model, both accuracy and energy efficiency were evaluated using comparative analyses on the AUTSL and WLASL datasets. Notably, our single-modality approach demonstrated superior performance on the AUTSL dataset compared to other existing methods. Furthermore, our multimodal approach achieved results that were very competitive with other state-of-the-art approaches, underscoring its efficacy in handling complex SLR tasks.

#### 4.3.1. Comparison Experiment on AUTSL Dataset

Table 2 provides a comprehensive comparison of our sign language recognition (SLR) methods against state-of-the-art techniques on the AUTSL dataset, categorizing the comparisons between unimodal and multimodal approaches.

**Unimodal-Based Methods Comparison:** In the unimodal category, our comparisons include baseline methods such as BLSTM [50], VTN-PF [52], SL-TSSI-DenseNet [53], MS-G3D [54], TMS-Net [55], and the two unimodal methods provided by SamSLR-V1 [57] and SamSLR-V2 [40]. It can be seen that skeleton-based methods generally exhibit higher accuracy than those based solely on RGB images. Our enhanced ResNet2+1D method, which uses RGB images, achieves a Top-1 accuracy of 95.83% and a Top-5 accuracy of 99.73%, surpassing most other RGB-based methods. In addition, our skeleton-based ST-GCN method outperforms all other unimodal methods, with a Top-1 accuracy of 96.85% and a Top-5 accuracy of 99.84%.

**Multimodal-Based Methods Comparison:** For multimodal methods, we compare our method against a series of previous research projects, including SwinRSL [58], MS-G3D [54], NTIS [56], SamSLR-V1, and SamSLR-V2. Our multimodal SLR method, which integrates skeleton and RGB image data, shows superior performance with a Top-1 accuracy of 98.00% and a perfect Top-5 accuracy of 100%. However, the top-rated SamSLR-V2 method slightly leads with a Top-1 accuracy of 98.53% and a Top-5 accuracy of 100%. It is important to note that SamSLR-V2 employs multiple additional modalities including optical flow, depth, and HHA images, resulting in a more complex and resource-demanding model. In contrast, our approach uses only two modalities (RGB and skeleton), which provides a more efficient yet competitive solution in terms of both accuracy and computational cost. Therefore, the slightly lower accuracy compared to SamSLR-V2 is balanced by the reduced complexity and improved inference speed of our method.

#### 4.3.2. Comparison Experiment on WLASL Dataset

Table 3 presents a detailed comparison of our sign language recognition (SLR) methods against current state-of-the-art techniques on the WLASL dataset, divided into evaluations based on single-modality and multimodality approaches. For the unimodal methods, which include baseline models WLASL and I3D [51], BSL [59], Slgtmorfer [60], and TMS-Net, our enhanced ResNet2+1D method based on RGB images has Top-1 accuracy and Top-5 accuracy of 48.67% and 83.45%, respectively, outperforming most other RGB image-based methods. The Top-1 accuracy and Top-5 accuracy of our skeleton-based ST-GCN method are 54.89% and 87.35%, respectively, surpassing most of the unimodal methods.

For the multimodal category, comparisons are made with methods including MSNN [61], BEST [62], Sign-BEST [63], and SamSLR-V2, where our multimodal SLR method has a Top-1 accuracy and Top-5 accuracy of 59.05% and 91.50%, respectively. These results show superior performance over MSNN and BEST but indicate there is still a gap when compared to the top performance of current state-of-the-art methods like SamSLR-V2.

While these results are encouraging, we acknowledge some limitations. Our evaluation primarily relies on accuracy metrics, and more comprehensive analyses including F1 scores and confusion matrices will be conducted in future work to better understand per-class performance and robustness. Furthermore, a systematic investigation of failure cases—such as low-resolution videos, rare or ambiguous gestures, and challenging backgrounds—was not performed in this study and remains a valuable direction for future research.

### 4.4. Ablation Study

For the ablation study, we evaluate the efficacy of four modalities.

#### 4.4.1. Effects of Multi Skeleton Features

We individually tested streams over joint features, joint motion features, bone features, and bone motion features using the MSGCN model as shown in Table 4 and obtained the following results: the Top-1 accuracy of each single skeleton feature on the AUTSL dataset was 95.64%, 96.02%, 93.99%, and 94.63%, respectively, whereas the Top-1 accuracy of the multi-stream skeleton feature was 96.85%, which is significantly higher than the results of the single skeleton feature.

On the WLASL dataset, the Top-1 accuracies for each single skeleton feature are 46.03%, 46.75%, 34.78%, and 34.90%, respectively, while the Top-1 accuracy for the multi-stream skeleton feature is 54.89%, which is a significant improvement compared to the single-skeleton modality. The enhanced performance of the multi-stream approach on both datasets suggests that a combined view of skeletal information offers a more complete understanding of the gestures in sign language. This holistic view allows the SLR model to more accurately capture the nuances of sign language, resulting in higher recognition accuracy.

The results from our ablation study confirm the effectiveness of multi-stream skeleton features in boosting SLR accuracy and highlight the advantages of multimodal feature fusion, especially in complex recognition tasks such as SLR. This strategy not only heightens accuracy but also emphasizes the importance of utilizing diverse data streams to develop robust and efficient recognition systems.

#### 4.4.2. Effects of Different Components in MSGCN

We conducted ablation studies on the AUTSL dataset to evaluate the effectiveness of each component within our proposed MSGCN. As shown in Table 5, the inclusion of the SJAM and SETCN modules significantly enhanced the model’s performance. The Top-1 accuracies are improved by 0.82%, 0.94%, 0.91%, and 1.74% for the individual skeleton features, with the most notable enhancement observed in the skeletal motion features. In addition, the Top-1 accuracy for multi-stream skeleton features improves by 0.59%.

#### 4.4.3. Effects of 3D Deformable Convolution

Further ablation experiments were conducted on the D-ResNet model. Based on results presented in Table 6, the original RGB image-based ResNet2+1D model from SAMSLR-V2 showed a Top-1 accuracy of 95.0%. By integrating deformable spatial and temporal convolutions in place of traditional convolutions, our improved model achieved a Top-1 accuracy of 95.83%, clearly surpassing the SAMSLR-V2’s ResNet2+1D model. This confirms the effectiveness of our modifications.

#### 4.4.4. Effects of Multimodal Fusion

Additionally, we conducted ablation experiments on our multimodal SLR method, which integrates both skeleton and RGB modalities. As shown in Table 6, this multimodal approach achieves substantial improvements over unimodal methods, with Top-1 accuracy increases of 1.43% and 2.25%, respectively. While RGB images are often affected by complex backgrounds that reduce recognition accuracy, skeleton data’s performance depends heavily on the precision of the skeleton detection model. Our multimodal fusion method effectively mitigates these individual limitations, significantly enhancing overall recognition performance.

We highlight the critical contributions of our key modules in achieving these results. The MSGCN module strengthens skeleton feature extraction by effectively capturing spatial-temporal dependencies through a multi-stream graph convolutional network. Meanwhile, the D-ResNet, leveraging deformable convolutions, adaptively models complex spatial and temporal variations in RGB videos, surpassing conventional 3D CNN approaches. Furthermore, the multimodal fusion module employs a gate-based mechanism to seamlessly integrate complementary information from both skeleton and RGB modalities, thereby improving the model’s robustness and recognition accuracy.

To evaluate the inference performance of our proposed model, we report the frames per second (FPS) for each skeleton stream and fusion model in Table 4 and for different backbones in Table 6. As shown, individual skeleton streams such as Joint and Bone maintain high inference speeds above 320 FPS, with Joint Motion reaching 355 FPS due to its relatively simple spatial features. The multi-stream fusion model, which integrates four types of skeleton features via a gated mechanism, still achieves a practical inference speed of 160 FPS.

Furthermore, when comparing backbone structures in Table 6, our multi-modal model that incorporates RGB and skeleton features maintains a speed of 70 FPS, which is sufficient for real-time applications. Despite involving richer modalities and more complex feature interactions, the model demonstrates an efficient trade-off between accuracy and inference latency.

These results highlight that our approach, while leveraging multi-stream fusion and cross-modal enhancement, remains computationally efficient and suitable for deployment in real-time or resource-constrained environments.

### 4.5. Limitation Discussion

The proposed approach still has some limitations. First, the experiments were conducted on only two datasets, AUTSL and WLASL, which mainly cover Turkish and American Sign Languages, respectively. Therefore, the cross-language generalizability of the model remains untested. Second, the evaluation metrics were limited to accuracy (Top-1/Top-5), without a more thorough assessment of other important indicators such as F1 scores, per-class accuracy, and confusion matrices. Third, for the multimodal fusion framework, we did not conduct a quantitative analysis of potential information redundancy in the fusion layers, which could affect the model’s efficiency and performance. Fourth, the pre-training strategy differed between modalities, as the RGB stream and skeleton stream used separate datasets for pre-training. This inconsistency may influence the balance and comparability of feature representations.

In future work, we plan to address these limitations by conducting cross-lingual transfer learning experiments on additional datasets in different sign languages, incorporating more comprehensive evaluation metrics, optimizing and analyzing the multimodal fusion process to reduce redundancy, and establishing a consistent pre-training scheme across modalities. These improvements will help enhance the generalizability, robustness, and interpretability of the proposed framework.

## 5. Discussion

Sign language is an important tool for hearing-impaired people to communicate with hearing people; therefore, SLR is a very important research field with broad application scenarios and application value. The research path of SLR is similar to action recognition, but the complexity and diversity of gesture movements make it more challenging than traditional action recognition through sensor networks.

In this paper, we introduce an innovative multimodal-based SLR framework to improve the accuracy of SLR. Specifically, we build a Multi-Stream Spatio-Temporal Graph Convolutional Network based on skeletons, integrating modules such as a decoupling graph convolutional network, a self-emphasizing temporal convolutional network, and a spatio-temporal joint attention module to enhance the model’s ability to recognise sign language actions. Based on RGB images, we build a 3D ResNet model based on deformable convolution to model complex spatial and temporal sequences in the original raw images. To take full advantage of the complementary information between the multi-modal signals, a gating mechanism-based multi-stream fusion module is employed to merge the results of the two modalities. Finally, we conduct a large number of comparative and ablation experiments on two public datasets, AUTSL and WLASL, to validate the effectiveness of the individual modules and also to demonstrate the validity and superiority of our method.

However, the current fusion mechanism may introduce redundancy when integrating multi-source information, which could be further optimized through more efficient modality alignment and complementary information fusion strategies. In addition, future work could explore cross-dataset and cross-lingual transfer learning strategies to enhance the generalization and adaptability of the model for multilingual sign language recognition tasks.

## Figures and Tables

**Table 1:** Summary of commonly used word-level sign language datasets in recent years.

Dataset	Samples	Classes	Language	Signers
WLASL	21,083	2000	American	119
AUTSL	36,302	226	Turkish	43
CSL-500	125,000	500	Chinese	50
LSA64	3200	64	Argentinian	10
BSL-1K	273,000	1064	British	40

**Table 2:** Comparison results on the AUTSL dataset, where R, S, F, and D denote RGB, skeleton, optical flow, and depth images, respectively. The best result is highlighted in bold.

Methods	Modalities	Top-1 (%)	Top-5 (%)
BLSTM [50]	R	49.22	-
VTN-PF [52]	R	92.92	-
SL-TSSI-DenseNet [53]	S	93.13	-
SamSLR-V2 (R) [40]	R	95.00	99.47
D-ResNet (Ours)	R	95.83	99.73
MS-G3D [54]	S	95.95	99.55
SamSLR-V2 (S) [40]	S	96.53	99.81
TMS-Net [55]	S	96.62	99.71
**MSGCN (Ours)**	**S**	**96.85**	**99.84**
MS-G3D [54]	R + S	96.15	-
NTIS [56]	R + S + O	96.37	-
SamSLR-V1 [57]	R + S + D + O	97.51	100
**SamSLR-V2** [40]	**R + S + D + O**	**98.53**	**100**
Ours	R + S	98.13	100

**Table 3:** Comparison results on WLASL dataset, where R, S, F, and D denote RGB, skeleton, optical flow, and depth images, respectively.

Methods	Modalities	Top-1 (%)	Top-5 (%)
WLASL [51]	S	22.54	49.81
I3D [51]	R	32.48	57.31
BSL [59]	R	46.82	79.36
SamSLR-V2 (R) [40]	R	47.51	80.30
D-ResNet (Ours)	R	48.67	83.45
SamSLR-V2 (S) [40]	S	51.50	84.94
Slgtformer [60]	S	47.42	79.58
**TMS-Net** [55]	**S**	**56.40**	**88.96**
MSGCN (Ours)	S	54.89	87.35
MSNN [61]	R + S + F	47.26	87.21
BEST [62]	R + S	54.59	88.08
Sign-BEST [63]	R + S	54.69	87.49
SamSLR-V1 [57]	R + S + D + O	58.73	91.64
**SamSLR-V2** [40]	**R + S + D + O**	**59.39**	**91.48**
Ours	R + S	59.05	91.50

**Table 4:** Results of multi-stream skeleton feature fusion ablation experiments on AUTSL and WLASL datasets.

Skeleton Feature	AUTSL		WLASL		FPS
	Top-1 (%)	Top-5 (%)	Top-1 (%)	Top-5 (%)	
Joint	95.64	99.65	46.04	78.86	320
Bone	96.02	99.60	46.75	79.45	322
Joint Motion	93.99	99.23	34.78	65.53	355
Bone Motion	94.63	99.41	34.90	66.47	335
Multi-Stream	96.85	99.84	54.89	87.35	160

**Table 5:** Results of multi-stream skeleton feature fusion ablation experiments under different backbones on AUTSL dataset.

Skeleton Feature	Decoupling GCN		SJAM		SETCN	
	Top-1 (%)	Top-5 (%)	Top-1 (%)	Top-5 (%)	Top-1 (%)	Top-5 (%)
Joint	94.82	99.36	95.08	99.55	95.64	99.65
Bone	95.08	99.31	95.64	99.52	96.02	99.60
Joint Motion	93.08	98.90	93.59	99.20	93.99	99.23
Bone Motion	92.89	98.96	93.93	99.33	94.63	99.41
**Multi-Stream**	**96.25**	**99.57**	**96.55**	**99.81**	**96.85**	**99.84**

**Table 6:** Results of multi-stream skeleton feature fusion ablation experiments under different backbones on AUTSL and WLASL datasets.

Models	AUTSL		WLASL		FPS
	Top-1 (%)	Top-5 (%)	Top-1 (%)	Top-5 (%)	
D-ResNet	95.83	99.73	48.67	83.45	90
MSGCN	96.85	99.84	54.89	87.35	160
Multimodal	98.13	100	59.05	91.50	70

## Data Availability

The data used in the experiment can be obtained at: https://chalearnlap.cvc.uab.cat/dataset/40/description (accessed on 1 October 2023) and https://dxli94.github.io/WLASL (accessed on 1 October 2023).
